# Novel Class of Proteasome Inhibitors: In Silico and In Vitro Evaluation of Diverse Chloro(trifluoromethyl)aziridines

**DOI:** 10.3390/ijms232012363

**Published:** 2022-10-15

**Authors:** Laura Ielo, Vincenzo Patamia, Andrea Citarella, Thomas Efferth, Nasim Shahhamzehei, Tanja Schirmeister, Claudio Stagno, Thierry Langer, Antonio Rescifina, Nicola Micale, Vittorio Pace

**Affiliations:** 1 Department of Chemistry, University of Turin, Via P. Giuria 7, 10125 Torino, Italy; 2 Dipartimento di Scienze del Farmaco e della Salute, University of Catania, Viale A. Doria 6, 95125 Catania, Italy; 3 Department of Chemical, Biological, Pharmaceutical and Environmental Sciences, University of Messina, Viale Ferdinando Stagno D’Alcontres 31, 98166 Messina, Italy; 4 Department of Pharmaceutical Biology, Institute of Pharmaceutical and Biomedical Sciences, Johannes Gutenberg University, Staudinger Weg 5, 55128 Mainz, Germany; 5 Department of Medicinal Chemistry, Institute of Pharmaceutical and Biomedical Sciences, Johannes Gutenberg University, Staudinger Weg 5, 55128 Mainz, Germany; 6 Department of Pharmaceutical Sciences, Division of Pharmaceutical Chemistry, University of Vienna, Josef-Holaubek-Platz 2, 1090 Vienna, Austria

**Keywords:** proteasome inhibitors, aziridines, computational studies, in vitro assays, anti-proliferative activity

## Abstract

The ubiquitin-proteasome pathway (UPP) is the major proteolytic system in the cytosol and nucleus of all eukaryotic cells. The role of proteasome inhibitors (PIs) as critical agents for regulating cancer cell death has been established. Aziridine derivatives are well-known alkylating agents employed against cancer. However, to the best of our knowledge, aziridine derivatives showing inhibitory activity towards proteasome have never been described before. Herein we report a new class of selective and nonPIs bearing an aziridine ring as a core structure. In vitro cell-based assays (two leukemia cell lines) also displayed anti-proliferative activity for some compounds. In silico studies indicated non-covalent binding mode and drug-likeness for these derivatives. Taken together, these results are promising for developing more potent PIs.

## 1. Introduction

**Figure 1:**
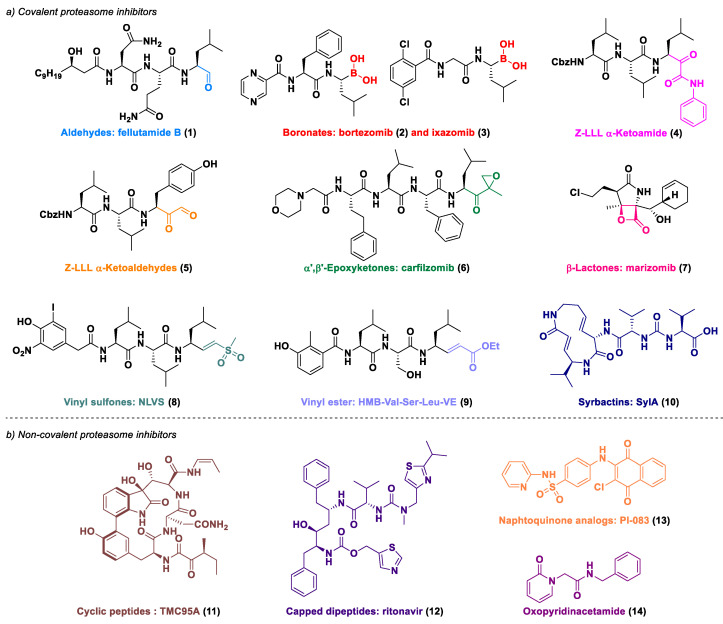
Representative examples of the major classes of covalent (**a**) and non-covalent (**b**) PIs from natural and synthetic sources.

The dynamic conditions of intracellular proteins are preserved by a perfect equilibrium between protein synthesis and degradation. Thus, the quality and quantity of proteins within a cell must be strictly regulated according to cellular needs or physiological demands. In this regard, the ubiquitin-proteasome pathway (UPP) constitutes the major proteolytic system in the cytosol and nucleus of all eukaryotic cells. It is crucial for maintaining intracellular protein homeostasis in physiological conditions and during adaptive stress responses [1,2,3,4,5]. UPP catalyzes the degradation of most short-lived and long-lived proteins, which comprehend the number of proteins in mammalian cells [6,7]. The degradation of proteins by the UPP is a cyclic pathway involving an initial step of ubiquitin (Ub) conjugation to the protein substrate, resulting in the degradation of the polyubiquitinated protein via the 26S proteasome complex [5,8,9,10]. The eukaryotic 26S proteasome is a large (~2.5 MDa) multifunctional particle, composed of a barrel-shaped 20S core particle (CP), which contains the protease subunits, capped by two 19S regulatory particles (RPs) that control the proteolytic function of the protease core. The 20S proteasome is the proteolytically active key component of the ubiquitin-proteasome system. It is constituted by four heptameric rings stacked in a α7β7β7α7 arrangement. The two inner β-rings contain the proteolytic active sites (β1–7). In particular, the three proteolytic subunits are β1, β2, and β5, which have distinct substrate specificities [11,12,13]. β5 subunits display “chymotrypsin-like” (ChT-L) activity and are mainly involved in protein degradation. Therefore, they emerged as the principal targets for developing efficacious anticancer agents [14,15]. The other two proteolytic subunits of the proteasome, β2 and β1, must be considered as co-targets of anticancer agents to achieve an efficient protein breakdown. β2 subunits possess “trypsin-like” (T-L) activity, whereas β1 subunits are referred to as “post-glutamate peptide hydrolase” (PGPH) or “caspase-like” (C-L) [13,16,17]. All three catalytic subunits contain an *N*-terminal residue, Thr1, of which the hydroxyl group acts as a nucleophile and interacts with the peptides of the proteins to be degraded [16,18,19]. Immediately after the disclosure of the UPP and its relevance to protein and cellular homeostasis, preclinical studies on the presumed role of proteasome inhibitors (PIs) as critical agents for regulating cancer cell death have commenced [20,21]. The proteasome was identified and validated as a pivotal target in protein quality control and turnover, cell differentiation, cell-cycle regulation, and apoptosis [5,8,9,10,22]. To date, three PIs have been approved by the US Food and Drug Administration Agency (FDA) and the European Medicine Agency (EMA), Velcade^®^ (bortezomib, **2**) [23,24], Ninlaro^®^ (ixazomib, **3**) [23], and Kyprolis^®^ (carfilzomib, **6**) [23] (Figure 1), as new drugs to treat multiple myeloma (MM) and mantle-cell lymphoma (MCL). It is known that the co-inhibition of the β5 subunits with either the β1 or the β2 subunits provides the maximal anti-tumor effect and represents the ideal profile for a drug candidate [25,26,27]. On the contrary, the inhibition of all proteasome subunits may develop cytotoxicity [28]. The majority of 20S PIs currently reported in literature [2,29,30,31], are peptide-based compounds, featured with a *C*-terminal electrophilic warhead that forms covalent adducts with the hydroxyl group of Thr1 in the active sites. A reversible mechanism of action is observed for aldehydes (**1**) [3,32,33,34], boronates (**2–3**) [23,24,35,36,37], α-keto-amides (**4**) [37,38], and α-keto-aldehydes (**5**) [39]. α’,β’-Epoxyketones (**6**) [3,34,40], β-lactones (**7**) [41,42], vinyl sulfones (**8**) [43,44,45], vinyl esters (**9**) [46], and syrabactins (**10**) [47] act as irreversible inhibitors (Figure 1a) [2]. For the latter, the covalent mode of action and the elevated reactivity of the compounds may often lead to off-target interactions. Lately, diverse new non-covalent PIs were identified (Figure 1b) [17,48,49,50,51,52]. Even if less widely investigated with respect to covalent inhibitors, they provide a valid and milder alternative mechanism for proteasome inhibition.

## 2. Results and Discussion

**Scheme 1:**
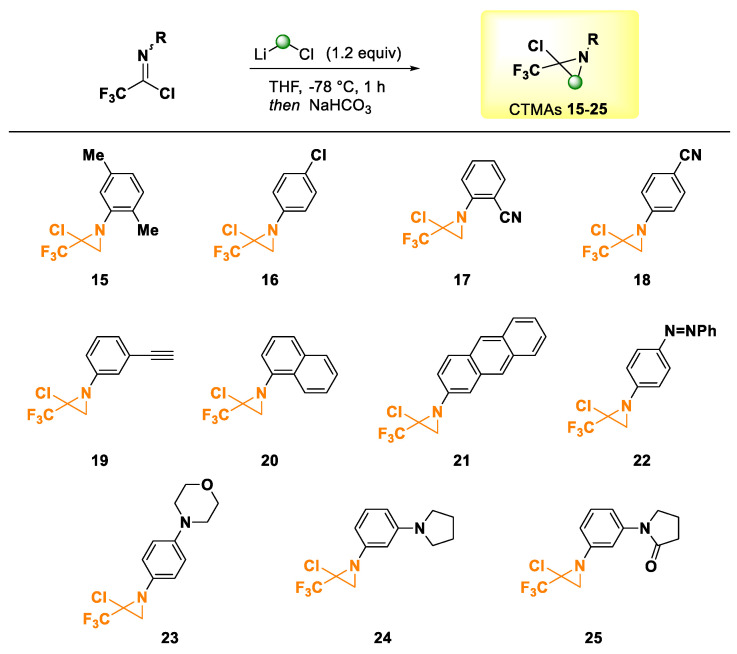
Chemical structure of chloro(trifluoromethyl)aziridines (CTMAs, **15**–**25**).

**Figure 2:**
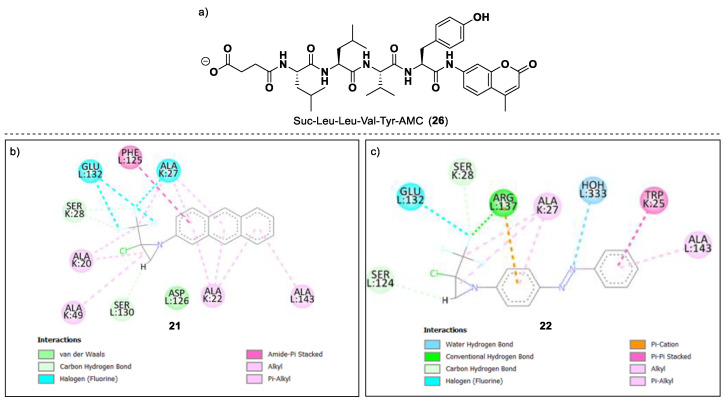
Reference compound structure Suc-Leu-Leu-Val-Tyr-AMC (**26**) is used as the positive control (**a**). The 2D poses of CTMAs **21** (**b**) and **22** (**c**), respectively, within the receptor pocket.

Despite the remarkable achievements in obtaining PIs, further research is still needed due to the lack of selectivity and resistance development of the inhibitors and drugs reported so far. Recently, we discovered a new class of aziridine featuring compounds as promising PIs. Albeit aziridine derivatives are well-known alkylating agents employed against cancer [53,54,55], to the best of our knowledge, aziridine derivatives showing inhibitory activity towards proteasome have never been reported. Thus, inspired by in silico predictions (see hereinafter), the in vitro biological activity of selected chloro(trifluoromethyl)aziridines (CTMAs) (Scheme 1), synthesized by Ielo et al. in 2019 [56], was evaluated toward human 20S proteasome. CTMAs were synthesized via homologation chemistry enriched with a brand-new interesting biological applicability in the field of drug discovery, as already evidenced by our research group activity [57,58,59,60]. The investigation started by performing docking studies using proteasome β5 subunit of *Saccharomyces cerevisiae* (PDB ID: 4HRD) [61] in order to assess possible binding modes of CTMAs to the catalytic pocket of the target. The peptide Suc-Leu-Leu-Val-Tyr-AMC (**26**, Figure 2a) was used as a reference proteasome substrate. Docking results predicted promising activities as PIs (Table 1). With these results in hand, we decided to evaluate the in vitro inhibitory activity of compounds **15**–**25** against the ChT-L activity of the human 20S proteasome [31,44,62,63,64,65,66]. The calculated free energies of binding (Δ*G*) and the calculated and experimental Ki values at the binding site of the proteasome are reported in Table 1.

Screening assays against human 20S proteasome ChT-L activity (β5 subunit) were performed for all CTMAs (**15**–**25**) at a concentration of 20 μM using DMSO (Dimethyl Sulfoxide) as a negative control [67]. The two most active CTMAs (i.e., **21** and **22**) showed inhibitory activity of 68% and 67%, respectively, suggesting that bulky hydrophobic groups at the aziridine nitrogen are preferred for effective binding to the target. Therefore, they were selected for continuous assays providing IC_50_ values of 13.6 and 14.1 μM, respectively, and binding affinity (Ki) in the low-micromolar range (Table 1).

Preliminary assays (i.e., screening at 20 μM) carried out on several cysteine and serine proteases, as well as on proteasome β1 and β2 subunits, did not show any inhibitory activity (inhibition range 0–5%), indicating a marked selectivity of compounds **15**–**25** towards proteasome β5 subunits. Afterwards, we moved to cell-based assays to assess a correlation between the proteasome ChT-L inhibition and the anti-proliferative activity of CTMAs by using the resazurin method on two different leukemia cells lines (Table 2) [68,69,70]. Derivatives **21** and **22** displayed the best anti-proliferative profile also in this assay with an IC_50_ value of 25.45 and 32.82 μM on CCRF-CEM (drug-sensitive acute lymphocytic leukemia cells) and of 24.08 and 67.72 μM on CEM/ADR500 (multidrug-resistant leukemia sub-cell line) [71,72,73], respectively. Noteworthy, both **21** and **22** didn’t show cytotoxicity against healthy peripheral blood mononuclear cells (PBMCs) (Table 2) (Figure S1).

In silico studies were performed using AutoDock Vina implemented in the YASARA software. The docking studies were performed on all the protonation states of the compounds at pH 7.4, previously calculated using the Marvin software. Following in silico studies, it was seen that the CTMAs tested can bind the receptor non-covalently and nevertheless possess a good inhibitory capacity. Considering CTMAs SAR (Structure-Activity Relationships), the phenyl ring substitution seems favorable in para-position since a decrease or even loss of activity was observed in derivatives substituted in ortho-position. Linear conjugated π systems, such as anthracene (**21**) and diazo-diphenyl (**22**), confer the best activity. The 2D structures for the two most active CTMAs, **21** and **22**, are shown in Figure 2b,c, respectively. CMTAs **21** and **22** establish somewhat similar interactions within the receptor site. From the 2D poses of the two compounds, it can be seen that both ligands establish hydrophobic interactions with the residues Ala27, Glu132, Ser28, and Ala143. The presence of a greater hydrophobic portion due to the anthracene moiety (**21**) allows the compound **21** to establish π-alkyl interactions with the Ala22 residue (Figure 2b). CTMA **22**, due to the presence of the two central nitrogen atoms, establishes a hydrogen bond (2.57 Å) with a water molecule inside the receptor site (Figure 2c). In addition, we performed in silico ADMET studies on CTMAs **21** and **22** to further strengthen the results of the docking studies. The ability to reach targets in bioactive form was evaluated using the SwissADME web platform (http://swissadme.ch, accessed on 15 July 2022). Notably, the technologies implemented in this platform can predict the false positive results commonly observed in biochemical small molecule assays with a fair degree of certainty. The two compounds simultaneously satisfy Lipinski’s [74] and Veber’s [75] rule for drug similarity. Both have a Bioavailability Score of 0.55. Importantly, CTMA **21** showed no alerts on the outcome of the PAINS model of pan assay interference structures [76], designed to exclude small molecules that might show false positives in bioassays.

**Figure 3:**
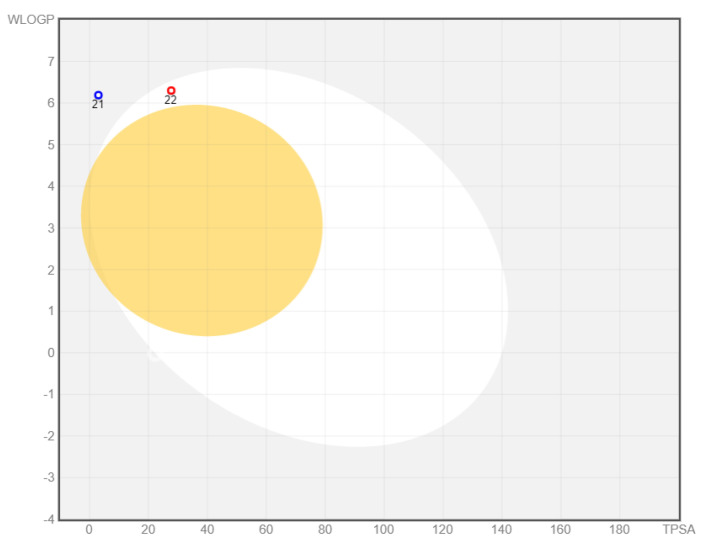
BOILED-Egg plot. Points located in the BOILED-Egg’s yolk (yellow) represent the molecules predicted to passively permeate through the blood-brain barrier, whereas the ones in the egg white are relative to the molecules predicted to be passively absorbed by the gastrointestinal tract; the blue dots (**21**) indicate the molecules for which it was expected to be effluated from the CNS by the P-glycoprotein, whereas the red ones (**22**) point to the molecules predicted not to be effluated from the CNS by the P-glycoprotein.

Human gastrointestinal uptake (HIA) and blood-brain barrier (BBB) penetration, related to absorption and distribution parameters, respectively, were graphically represented by the extended and revamped version of the Edan-Egg model, called the Brain Or IntestinaL EstimateD (BOILED) predictive permeation model (BOILED-Egg). Visual analysis of Figure 3 shows that CTMA **22** is passively absorbed by the gastrointestinal tract, and could be effluated from the central nervous system (CNS) with the aid of the P-glycoprotein. None of them was predicted to passively permeate through the BBB.

## 3. Materials and Methods

### 3.1. General Methods

Melting points were determined on a Reichert–Kofler hot-stage microscope and are uncorrected. Mass spectra were obtained on a Shimadzu QP 1000 instrument (EI, 70 eV) and on a Bruker maXis 4G instrument (ESI-TOF, HRMS). ^1^H, ^13^C, ^19^F, and ^15^N NMR spectra were recorded with a Bruker Avance III 400 spectrometer (400 MHz for ^1^H, 100 MHz for ^13^C, 377 MHz for ^19^F, 40 MHz for ^15^N) and with a Bruker DRX spectrometer (200 MHz for ^1^H, 50 MHz for ^13^C) at 297 K. The center of the solvent signal was used as an internal standard which was related to TMS with *δ* 7.26 ppm (^1^H in CDCl_3_), *δ* 77.00 ppm (^13^C in CDCl_3_). ^15^N NMR spectra were referenced against external nitromethane (0.0 ppm), ^19^F NMR spectra by absolute referencing via ∈ ratio. Spin-spin coupling constants (J) are given in Hz. In nearly all cases, complete and unambiguous assignment of all resonances was performed by applying standard NMR techniques, such as APT, HSQC, HMBC, COSY, and NOESY experiments.

THF was distilled over Na/benzophenone. Chemicals were purchased from Sigma-Aldrich, Acros, Alfa Aesar, Fluorochem, and TCI Europe, otherwise specified. Organolithium reagents were kindly provided by Albemarle Corporation. Solutions were evaporated under reduced pressure with a rotary evaporator. TLC was carried out on aluminum sheets precoated with silica gel 60F254 (Macherey-Nagel, Merck, Darmstadt, Germany); the spots were visualized under UV light (λ = 254 nm) and/or KMnO_4_ (aq.) was used as a revealing system. Neutral Aluminium Oxide-Brockmann grade 2 (Alox-BG2) for chromatographic purifications was prepared as we previously reported [77]. MeLi-LiBr (1.5 M ethereal solution) was titrated immediately prior to use, according to the literature [78].

### 3.2. General Procedures

#### 3.2.1. General Procedure for Preparing Trifluoroacetimidoyl Chlorides [79]

To a solution of Ph_3_P (3.0 equiv) in DCE was added CCl_4_ (4.0 equiv), Et_3_N (1.2 equiv), and TFA (1.0 equiv) at 0 °C and the mixture was stirred for 10 min. After the solution was cooled to room temperature, suitable aniline (1.0 equiv) was added. The mixture was then refluxed overnight. The solvent was removed under reduced pressure, and the residue was diluted and washed with *n*-hexane several times and filtered. The filtrate was concentrated under reduced pressure, and the so-obtained crude mixture was subjected to chromatography (silica gel) to afford pure compounds.

#### 3.2.2. General Procedure for Preparing Chloro-Trifluoromethylaziridines (CTMAs) [56]

To a cooled (−78 °C) solution of trifluoromethylchloroimidate (1.0 equiv) in dry THF was added chloroiodomethane (1.3 equiv). After 2 min, an ethereal solution of MeLi-LiBr (1.2 equiv, 1.5 M) was added dropwise using a syringe pump (flow: 0.200 mL/min). The resulting solution was stirred for 1 h. Then 10% aq solution NaHCO_3_ (2 mL/mmol substrate) was added, and the reaction mixture was extracted with Et_2_O (2 × 5 mL) and washed with water (5 mL) and brine (10 mL). The organic phase was dried over anhydrous Na_2_SO_4_, filtered, and, after removal of the solvent under reduced pressure, the so-obtained crude mixture was subjected to chromatography (Alox-BG2) to afford pure compounds.

### 3.3. In Silico Studies

The studied molecules were drawn using Marvin Sketch and minimized toward molecular mechanics by Merck molecular force field (MMFF94) optimization using the Marvin Sketch geometrical descriptors plugin. The protonation states of the molecules were calculated assuming a 7.4 pH. The MMFF91 obtained 3D structures were subsequently optimized using the parameterized model number 6 semi-empirical Hamiltonian with the corrections to hydrogen bonding and dispersion (PM6-D3H4) implemented in the MOPAC package (vMOPAC2016). Docking calculations were made using AutoDock Vina, as implemented in the YASARA package, with the default docking parameters. The X-ray crystal structures of the co-crystal proteasome subunit β5/Carmaphycin B (PDB ID: 4HRD) were downloaded from the Protein Data Bank (www.rcsb.org). Only chains K and L were used. Water molecules were also removed. All amino acid residues were kept rigid, whereas all single bonds of ligands were treated as fully flexible for both proteins. A 10 Å simulation cell around all atoms of the co-crystallized ligand was used. AMBER 14 force field was used for the simulation.

### 3.4. Biological Assays

#### 3.4.1. Inhibition Assay for the Chymotrypsin-like Activity of the 20S Proteasome

The inhibitory activity of the compounds was evaluated by a standard fluorometric method [67]. Human 20S proteasome was obtained from a commercial source (Biomol GmbH, Hamburg, Germany), as well as the peptidic substrate (Bachem, Bubendorf, Switzerland) Suc-Leu-Leu-Val-Tyr-AMC·HCl for the chymotrypsin-like (ChT-L) activity of the enzyme. The proteolytic activity of the 20S proteasome was measured by monitoring the hydrolysis of the substrate by detecting the fluorescence of the product released, 7-amino-4-methyl coumarin (7-AMC), by means of an Infinite 200 PRO microplate reader (Tecan, Männedorf, Switzerland) at 30 °C with a 380 nm excitation filter and a 460 nm emission filter. Human 20S proteasome was employed for testing at a final concentration of 0.004 mg·mL^−1^ together with the fluorogenic substrate (100 μM) and compounds present at 20 μM (screening assay) or at variable concentrations (continuous assay). DMSO was used as a negative control. The reaction buffer consisted of 50 mM Tris pH 7.5, 10 mM NaCl, 25 mM KCl, 1 mM MgCl_2_·6H_2_O, 0.03% SDS and 5% DMSO. The compounds and enzyme were incubated for 30 min at 30 °C prior to substrate addition. Product release from substrate hydrolysis was monitored continuously for 10 min.

#### 3.4.2. Inhibition Assay for the Post-Glutamyl Peptide Hydrolyzing (PGPH or Caspase-like) Activity of the 20S Proteasome

The assay against the caspase-like (Casp-L) activity of the human 20S proteasome was performed in the same experimental conditions as for the ChT-L activity. The enzyme employed for testing was incubated at a final concentration of 0.003 mg·mL^−1^ together with the appropriate fluorogenic substrate Z-Leu-Leu-Glu-AMC·HCl (80 μM) and compounds present at 20 μM (screening assay) or at variable concentrations (continuous assay). DMSO was used as a negative control. The reaction buffer consisted of 50 mM Tris pH 7.5, 10 mM NaCl, 25 mM KCl, 1 mM MgCl_2_·6H_2_O, 0.03% SDS and 5% DMSO.

#### 3.4.3. Inhibition Assay for Trypsin-like Activity of the 20S Proteasome

The assay against the trypsin-like (T-L) activity of the human 20S proteasome was performed in the same experimental conditions as for the ChT-L and Casp-L activity. The enzyme employed for testing was incubated at a final concentration of 0.0025 mg·mL^−1^ together with the appropriate fluorogenic substrate Boc-Leu-Arg-Arg-AMC·HCl (85 μM) and compounds present at 20 μM (screening assay) or at variable concentrations (continuous assay). DMSO was used as a negative control. The reaction buffer consisted of 50 mM Tris pH 7.4, 50 mM NaCl, 0.5 mM EDTA, 0.03% SDS and 5% DMSO.

#### 3.4.4. Cell Culture

Drug-sensitive CCRF-CEM and multidrug-resistant (MDR) P-glycoprotein (P-gp)-over-expressing CEM/ADR5000 leukemic cells were kindly provided by Prof. Axel Sauerbrey (Department of Pediatrics, University of Jena, Germany). Cells were cultured in RPMI 1640 medium, including 10% fetal bovine serum (FBS) and 1% penicillin (1000 U mL^−1^)/streptomycin (100 mg mL^−1^) (Life Technologies, Darmstadt, Germany). Doxorubicin (5000 ng mL^−1^) was added to the culture medium to maintain overexpression of P-gp (MDR1/ABCB1) in CEM/ADR5000 cells every 14 days. The MDR-phenotype of CEM/ADR5000 cells has been characterized [71,72,73]. In vitro antiproliferative activity against CCRF-CEM and CEM/ADR5000 cell lines. The cytotoxic effects of the compounds in dimethyl sulfoxide (DMSO) were tested using a resazurin assay [68,69]. This assay is based on the reduction of the indicator dye, resazurin, to the highly fluorescent resorufin by viable cells [70]. The aliquot of 1 × 10^4^ cells per mL cells was seeded into 96-well plates and immediately treated with various concentrations of each compound. After 72 h of incubation at 37 °C, 20 µL resazurin 0.01% w/v solution was added to each well, and the plates were maintained at 37 °C for 4 h. Fluorescence was measured using an Infinite M2000 Pro-plate reader (Tecan, Crailsheim, Germany) with an excitation wavelength of 544 nm and an emission wavelength of 590 nm. Each experiment was done at least three times with six replicates each. The viability was analyzed in comparison with the untreated cells. Fifty percent inhibition (IC_50_) values are the drug concentrations required to inhibit 50% of cell proliferation and were calculated from a calibration curve by linear regression using Microsoft Excel.

## 4. Conclusions

In conclusion, a new class of non-covalent, selective, and not cytotoxic PIs has been described. The reported in silico and in vitro studies indicated the presence of promising derivatives within this class of compounds, leading the way for further optimization in order to develop more potent inhibitors.

## Figures and Tables

**Table 1:** Docking scores, predicted *K*_i_ on proteasome β5 subunit of *Saccharomyces Cerevisiae* (PDB ID: 4HRD), and inhibitory activity of compounds **15**–**25** compared to that of reference **26**.

Compound	Δ*G* Vina	*K*_i_ Calc. (μM)	ChT-L Activity (% Inhibition at 20 μM) ^1^	IC_50_/*K*_i_ (μM) ChT-L ^2^
**15**	−6.91	8.56	<20	-
**16**	−7.18	5.42	25 ± 1.1	-
**17**	−7.07	6.53	<20	-
**18**	−7.64	2.49	37 ± 0.5	-
**19**	−7.40	3.74	<20	-
**20**	−7.89	1.64	<20	-
**21**	−8.31	0.80	68 ± 0.4	13.6 ± 1.1/1.6 ± 0.13
**22**	−8.59	0.50	67 ± 0.1	14.1 ± 0.7/1.6 ± 0.08
**23**	−7.88	1.66	41 ± 1.2	-
**24**	−7.85	1.75	27 ± 0.3	-
**25**	−8.11	1.31	34 ± 1.4	-
**26**	−8.75	0.38	-	-

^1^ Screening assays on human 20S proteasome ChT-L activity (β5 subunit). ^2^ Continuous assays on human 20S proteasome ChT-L activity (β5 subunit) with final inhibitor concentrations 0, 2.5, 5, 10, 20, 30, 40, and 50 μM performed for 30 min only for compounds that showed >60% of inhibition in the screening test. IC_50_ values include standard deviation from two independent experiments, each performed in duplicate. *K*_i_ ± SD values have been calculated using the Cheng-Prusoff equation. The *K*_m_ values were determined in separate experiments: For ChT-L activity with Suc-Leu-Leu-Val-Tyr-AMC *K*_m_ = 13 μM.

**Table 2:** Cytotoxicity of compounds **15**–**25** towards drug-sensitive acute lymphocytic leukemia cells (CCRF-CEM), multidrug-resistant leukemia sub-cell line (CEM/ADR5000), and healthy peripheral blood mononuclear cells (PBMCs) using the resazurin reduction assay. All values are shown as mean ± standard deviation (SD) of three independent experiments. The degree of resistance was calculated by dividing the IC_50_ value of resistant by that of sensitive cells.

Compound	CCRF-CEM IC_50_ (µM)	CEM/ADR5000 IC_50_ (µM)	PBMCs CC_50_ (µM)	Degree of Resistance
**15**	>100	>100	-	-
**16**	55.03 ± 1.33	>100	-	-
**17**	>100	>100	-	-
**18**	>100	>100	-	-
**19**	>100	>100	-	-
**20**	>100	>100	-	-
**21**	25.45 ± 4.08	24.08 ± 4.05	>100	0.95
**22**	32.82 ± 1.28	67.72 ± 8.80	>100	2.06
**23**	56.95 ± 2.20	>100	-	-
**24**	42.15 ± 1.86	>100	-	-
**25**	82.41 ± 3.50	67.97 ± 3.20	-	0.82
**Doxorubicin**	0.044 ± 0.01	20.50 ± 2.59	-	463.90

## Data Availability

The data that support the findings of this study are available from the corresponding authors upon reasonable request.

## Supplementary Materials

The following supporting information can be downloaded at: https://www.mdpi.com/article/10.3390/ijms232012363/s1.
