# Supplementary material for: Novel Class of Proteasome Inhibitors: In Silico and In Vitro Evaluation of Diverse Chloro(trifluoromethyl)aziridines
Source: Int J Mol Sci. 2022 Oct 15;23(20):12363. doi: 10.3390/ijms232012363 (PMC9603864; doi:10.3390/ijms232012363)

Figure S1. Dose-response curves of compound **21**(A) and compound **22**(B) against CCRF-CEM, CEM/ADR5000 cell lines, and PBMCs.

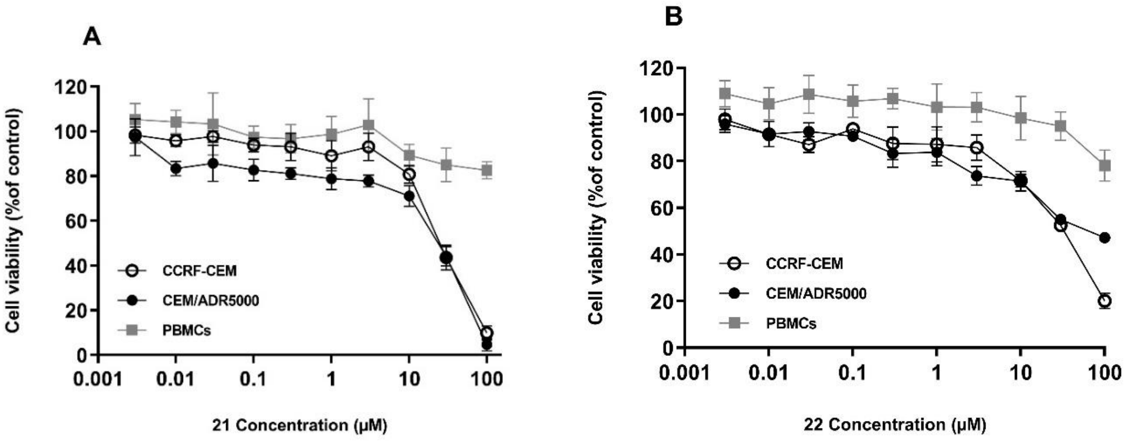

Supplement: Supplementary file 1 [file ijms-23-12363-s001.zip › ijms-1939667-supplementary.pdf]
